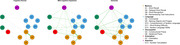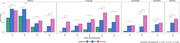# Differential network connectivity across Alzheimer’s disease stages: Insights from ADAS‐Cog

**DOI:** 10.1002/alz.095227

**Published:** 2025-01-09

**Authors:** Lucía Muñoz‐Gil, Laura Hernández‐Lorenzo, Jordi A Matias‐Guiu, Bojan Mihaljevic, José Luis Ayala

**Affiliations:** ^1^ Computer Science Faculty, Complutense University of Madrid, Madrid Spain; ^2^ Hospital Clinico San Carlos, San Carlos Health Research Institute, Madrid Spain; ^3^ Department of Artificial Intelligence, Technical University of Madrid, Boadilla del Monte Spain

## Abstract

**Background:**

Network neuropsychology is an emergent field dedicated to analyzing cognitive functions as interconnected systems. Although previous studies have explored cognitive network reorganization across the Alzheimer’s disease (AD) continuum using comprehensive neuropsychological batteries, these approaches often overlook the potential of single screening tests used in routine clinical practice. This study innovatively applies graphical models to data from isolated neurocognitive tests, specifically the Alzheimer’s Disease Assessment Scale‐Cognitive Subscale (ADAS‐Cog), to construct cognitive networks. Our approach not only handles the challenges posed by the non‐normal, ordinal and discrete nature of test data but also provides a more detailed understanding of the AD continuum.

**Method:**

We utilized ADAS‐Cog neuropsychological test scores from the Alzheimer’s Disease Neuroimaging Initiative (ADNI) database, categorizing subjects into Cognitively Normal (CN, n = 662), Mild Cognitive Impairment (MCI, n = 705), and Dementia (n = 219). To construct cognitive networks for each group, we calculated Spearman’s correlations and applied BIC graphical LASSO regularization, quantifying network stability through non‐parametric and case‐drop bootstrapping. We then rigorously assessed differences in network structures across the disease spectrum using the Network Comparison Test, focusing on several key graph metrics.

**Result:**

Our global connectivity analysis uncovered distinct variations in network densities across the AD spectrum, with notably higher density in the Dementia network. The most significant differences in node strengths were identified between the CN and Dementia groups, reinforcing ADAS‐Cog’s effectiveness in detecting various stages of Dementia. Furthermore, strength centrality analysis highlighted the growing relevance of ideational praxis, linked to the Visuospatial domain, in network reorganization across the AD continuum. In the Dementia networks, Immediate Memory and Language items emerged as predominant influencers, whereas Delayed Memory, Attention and Orientation were significantly more central in the MCI network.

**Conclusion:**

Our research highlights that dynamic changes in cognitive networks across the AD continuum can be effectively mapped using isolated neurocognitive tests like the ADAS‐Cog. Notably, the Dementia network emphasized the critical roles of Immediate Memory, Language and Visuospatial functions. Conversely, the MCI network revealed pronounced impairments in Orientation and Attention. These insights demonstrate the capacity of streamlined cognitive assessments to identify key cognitive changes, offering a quicker, yet robust alternative for monitoring disease progression in clinical settings.